# A Lightweight Hybrid Authentication and Key Agreement Protocol for Decentralized Device-to-Device Communication with Post-Quantum Confidentiality

**DOI:** 10.3390/s26103040

**Published:** 2026-05-12

**Authors:** Asday Savón-Berenguer, Sorin-Aurel Moraru, Juan Carlos García-Naranjo, An Braeken

**Affiliations:** 1 Department of Automatics and Information Technology, Transilvania University of Brasov (UnitBv), 500036 Brasov, Romania; asday.berenguer@unitbv.ro (A.S.-B.); smoraru@unitbv.ro (S.-A.M.); 2 Centre of Medical Biophysics, University of Oriente, Santiago de Cuba 90500, Cuba; jcnaranjo@uo.edu.cu; 3 Department of Electronics and Informatics (ETRO), Vrije Universiteit Brussel (VUB), 1050 Brussels, Belgium

**Keywords:** post-quantum, authentication and key agreement protocol, device-to-device, IPFS

## Abstract

Device-to-device (D2D) communication is expected to become a key component of 6G and IoT systems, enabling low-latency and infrastructure-independent connectivity. A major challenge is to establish secure session keys between previously unknown devices without relying on an online trusted third party, while also ensuring resilience against future quantum adversaries. This paper proposes a lightweight hybrid authentication and key agreement protocol for decentralized D2D communication. The approach combines IPFS-assisted distributed key discovery with a two-message protocol that uses post-quantum key encapsulation for long-term confidentiality, while retaining elliptic curve cryptography (ECC) for efficient real-time authentication under classical security assumptions.This design reflects the different temporal security requirements of confidentiality and authentication and provides a practical trade-off between quantum resilience and computational efficiency. The proposed scheme achieves mutual authentication under classical ECC assumptions, secure session key establishment, and resistance against common attacks, while providing post-quantum confidentiality protection against future quantum adversaries and removing the need for an online trusted third party (TTP) during protocol execution. The results demonstrate that the protocol offers a competitive and practical solution for secure decentralized D2D communication in IoT and future 6G environments.

## 1. Introduction

The increasing adoption of 5G and the emergence of 6G networks enable new paradigms in wireless communication, among which device-to-device (D2D) communication plays a central role. Unlike traditional cellular communication, which relies on a centralized infrastructure, D2D communication allows devices to communicate directly with each other, thereby reducing latency, improving spectral efficiency, and enabling new applications, such as autonomous systems, smart city infrastructures, and large-scale Internet of Things (IoT) deployments. In addition, D2D communication enhances network resilience by supporting operation in infrastructure-limited or disrupted environments.

A fundamental challenge in D2D communication is the establishment of secure session keys between devices that have no prior trust relationship and cannot rely on an online TTP during protocol execution. Existing approaches address this problem in different ways, each with inherent limitations. Physical-layer or device-specific characteristics, such as radio frequency fingerprints or environmental features, can be exploited to derive shared keys, but these approaches often require specialized hardware or complex machine learning models, making them impractical for constrained IoT devices [1].

Out-of-band mechanisms, including QR codes, near-field communication (NFC), or Bluetooth Low Energy (BLE), provide strong authentication guarantees through physical proximity, but they require manual interaction or additional communication channels, which limits scalability in automated environments [2]. Pre-shared key approaches eliminate the need for real-time key establishment but lack flexibility and scalability, especially in dynamic environments where devices encounter previously unknown peers.

Public key-based solutions, typically relying on certificates or public key infrastructures (PKIs), provide strong authentication but introduce significant management overhead. This issue becomes more pronounced in the post-quantum (PQ) setting, where public keys and signatures are substantially larger than their classical counterparts [3]. Identity-based cryptography (IBC) can reduce certificate overhead but introduces key escrow problems due to the reliance on a trusted authority [4].

To overcome these limitations, recent research has explored the use of distributed ledger technologies for decentralized key management. In such approaches, public key material is stored in a distributed and tamper-resistant manner, enabling devices to retrieve each other’s credentials without relying on centralized infrastructures [5,6]. However, existing works either focus primarily on the ledger infrastructure itself or rely on classical cryptographic mechanisms, without addressing the combined challenges of decentralized trust, PQ security, and lightweight D2D authentication in resource-constrained environments.

In this paper, we propose a lightweight hybrid authentication and key agreement (AKA) protocol for decentralized D2D communication. The proposed approach combines a distributed ledger-assisted key discovery mechanism with an efficient two-message authenticated key-establishment protocol. Public key material and device metadata are registered during a controlled onboarding phase and stored in an interplanetary file system (IPFS)-based distributed system, enabling decentralized lookup at runtime without requiring an online TTP. This design explicitly decouples trust establishment from runtime authentication. To the best of our knowledge, this is the first work that jointly achieves decentralized key discovery, PQ confidentiality, and lightweight authentication in a D2D setting without an online TTP.

At the cryptographic level, the protocol adopts a hybrid design in which PQ key encapsulation mechanisms (KEMs) are used to ensure long-term confidentiality of the established session keys, while elliptic curve cryptography (ECC) is retained for authentication. This choice is motivated by the different temporal security requirements of confidentiality and authentication: while confidentiality must remain secure against future quantum adversaries (harvest-now-decrypt-later attacks), authentication is verified in real time and can therefore rely on efficient classical mechanisms under the current threat model. This enables a practical trade-off between quantum resilience and computational efficiency, particularly for resource-constrained IoT devices.

The main contributions of this paper are as follows:

- We propose a decentralized D2D authentication and key agreement protocol without reliance on an online trusted third party.
- We introduce a hybrid cryptographic design combining post-quantum KEMs for confidentiality and ECC-based authentication for efficiency.
- We provide a formal security analysis in the ROR model and an informal analysis covering standard attack vectors.
- We evaluate the performance of the protocol and compare it with recent IoT and D2D authentication schemes.

The remainder of this paper is organized as follows. Section 2 reviews related work on D2D authentication and distributed ledger-based key management. Section 3 introduces the system architecture, security model, and notations. Section 4 presents the proposed protocol. Section 5 provides the security analysis, followed by implementation and performance evaluation in Section 6. Finally, Section 7 concludes this paper.

## 2. Related Work

We distinguish two types of schemes related to D2D communication, with and without distributed ledger technology.

### 2.1. Related Work on D2D Without DLT

In the literature, we distinguish the schemes with the presence of an online TTP acting as an intermediate party and without. Let us summarize some relevant contributions in both categories.

First, for the schemes with an intermediate party, in some of the cases, this party is not capable of deriving the resulting session key, while in others, the intermediate party plays an elementary role in the determination of the session key. In [7], a key agreement protocol has been designed for two IoT devices without any pre-existing trust relationship, utilizing only symmetric key-based operations, and relying on a server or proxy-based approach. The proxy is tasked with verifying the authentication and facilitating the key agreement between the IoT devices, while being unable to derive the established session key. The first variant of the scheme operates without requiring interactive input from the key distribution center to the proxy, but is vulnerable if both a compromised user and the proxy collude. The second variant is collision-resistant but necessitates an interactive key distribution center. A key agreement scheme between two devices with a server in between acting as an intermediate party but not capable of deriving the final session key, based on elliptic curve (EC) operations and physical unclonable functions (PUFs), has been proposed in [8] as an improvement of [9].

The 5G-AKA (authentication and key agreement) schemes [10], where a shared key is established between the user and serving network (SN), can be seen as a D2D key agreement protocol, but where the TTP, being the home network (HN), has full control over the derived session key. There are many variants presented based on the same architecture, being 5G-AKA schemes resisting malicious SNs [11], existing solely of symmetric key-based operations [12,13], and including PQ operations [14,15,16]. Also the scheme of [17] fits in this category, where first a token is generated by the HN and later used in the AKA protocol, using the NIST standard Kyber, between the devices.

For systems without a TTP, we limit the description to the PQ schemes. In [18], a survey is given on quantum-secure AKA protocols for IoT-enabled applications, and a distinction is made between schemes with two and three entities. From the identified schemes with two identities, we see that many of them [19,20,21,22,23] represent a client–server architecture and not a D2D architecture, where both devices are supposed to have no shared key material in advance. Among these schemes, ref. [19] relies on a code-based approach, ref. [20] is built upon the hardness of the shortest vector problem (SVP) and NTRU, [21] on the Ring Learning With Error (RLWE) problem, and [22,23] on the Inhomogeneous Short Integer Solution (ISIS) problem. In [24] (relying on LWE and ISIS) [25] (relying on Module Learning with Rounding), the public key material is known in advance or shared together with a certificate upon the start of the protocol. Practical attacks have been found against many of the ISIS-based AKA schemes in [26]. In other schemes [27,28] (both relying on RLWE), a common shared key is established based on ephemeral key material, and thus, no authentication of the legitimacy of the party is made.

Recent lightweight PQ-based schemes for IoT systems combine KEMs with classical cryptographic primitives to reduce computational overhead. For example, hybrid approaches integrating ECC, PQ KEM, and IPFS-assisted storage have been proposed for healthcare IoT environments [29]. However, this solution relies on fog or edge infrastructure and does not support fully decentralized authentication between previously unknown devices without online trusted entities. Another recent PQ AKA framework is presented in [30], where a multifactor approach is adopted combining ECC, physical unclonable functions (PUFs), and PQ KEMs. The protocol is evaluated on lightweight platforms, such as Raspberry Pi, and focuses on integrating multiple authentication factors to enhance security. However, the scheme assumes a structured environment with pre-established trust relationships and does not explicitly address decentralized D2D communication without a TTP. In particular, it does not consider the use of DLT for public key management, which limits its applicability in fully autonomous D2D settings.

While these works provide valuable insights into D2D authentication and key agreement, they exhibit several limitations. Many schemes rely on either a trusted intermediary during protocol execution or assume pre-shared key material between devices, which restricts scalability in dynamic environments. Moreover, several PQ schemes focus on client–server architectures rather than fully decentralized D2D settings, or do not provide explicit authentication guarantees between previously unknown devices. As a result, achieving lightweight, PQ-secure, and fully decentralized D2D authentication without an online trusted party remains an open challenge.

### 2.2. Related Work on D2D with DLT

The use of distributed ledger technology or the blockchain in general to facilitate or manage security in IoT environments is well established. For instance, in [31], a blockchain-enabled proxy re-encryption scheme is proposed. In [32], a blockchain-regulated automatic key refreshment mechanism for IoT systems has been developed, in which users are able to publicly verify the freshness of the security keys in use. The use of the blockchain to manage the certification process has been proposed in [33,34]. In [33], lightweight Elliptic Curve Qu Vanstone (ECQV) certificates are used, while in [34], a dedicated format of certificates has been proposed. However, neither scheme goes beyond proposing the blockchain structure and does not focus on a security protocol with advanced security strength. Also in [35], the blockchain is used to register legitimate devices to participate in D2D communication, but the key agreement process is based on physical characteristics. A blockchain-enforced cross-domain private-protected AKA scheme supporting attribute-based access control has been proposed in [36]. Unfortunately, the scheme is still based on classical cryptographic mechanisms. A more extended survey on distributed key management systems can be found in [37].

Recent studies have also highlighted the growing importance of integrating PQ cryptography with distributed and blockchain-based systems. A recent survey [38] shows that while hybrid and blockchain-based security solutions dominate the current landscape, fully PQ-secure and decentralized authentication mechanisms remain relatively underexplored. In particular, the combination of decentralized key discovery, PQ confidentiality, and lightweight D2D authentication without online trusted parties has not been sufficiently addressed in existing works.

In the broader context of secure vehicular and decentralized communication systems, recent works have also explored blockchain-assisted conditional anonymous authentication [39], adaptive tree-based group key agreement for vehicular ad hoc networks (VANETs) [40], and conditional privacy-preserving batch-verification authentication schemes for Internet of Vehicles deployment [41]. These approaches mainly focus on scalable authentication, anonymity, or group-oriented vehicular communication. In contrast, the present work specifically targets lightweight decentralized D2D authentication with post-quantum confidentiality and without reliance on an online trusted third party.

Although these approaches demonstrate the potential of DLT for key management and authentication, they primarily focus on certificate management, access control, or infrastructure-level mechanisms. They do not address the design of lightweight AKA protocols that combine decentralized key discovery with PQ security in a D2D setting. In particular, the integration of DLT-based key availability with efficient, two-party, PQ-secure key establishment remains largely unexplored.

### 2.3. Research Gap and Motivation

To highlight the differences between existing approaches and the proposed scheme, Table 1 summarizes key characteristics of representative solutions. As shown in Table 1, none of the existing schemes simultaneously achieves decentralized operation, the absence of pre-shared context, and PQ confidentiality. To the best of our knowledge, the proposed scheme is among the first to jointly achieve these properties while maintaining practical efficiency.

## 3. Preliminaries

### 3.1. System and Network Model

We consider a decentralized D2D communication environment consisting of a set of devices $D=\{D_{1},D_{2},\ldots \}$ that wish to establish secure communication channels without relying on an online TTP during protocol execution.

Each device $D_{i}\in D$ is initialized during an onboarding phase, in which its identity $ID_{i}$ and associated public key material are registered in a distributed storage system. This process is controlled by a trusted authority, which verifies the legitimacy of devices before allowing them to participate in the system. After onboarding, no online TTP is involved in the AKA process.

The distributed storage layer is realized using a decentralized system, such as IPFS, which acts as a publicly accessible directory storing device-related information. Each device $D_{i}$ is associated with a unique content identifier $CID_{i}$ that references its public key material and metadata. The corresponding CID values are obtained through the onboarding process, trusted registries, or deployment-specific authenticated discovery mechanisms. Therefore, devices treat the CID as a trusted reference to onboarding-approved public key material rather than as dynamically learned untrusted metadata. We assume that the IPFS layer ensures data integrity through content addressing, while authenticity is guaranteed by the onboarding process and optional authority signatures.

At runtime, two devices $D_{1}$ and $D_{2}$ establish a secure session through a two-message AKA protocol, using the public key information retrieved from the distributed storage.

### 3.2. Cryptographic Primitives

The proposed protocol relies on three classes of cryptographic primitives.

Symmetric encryption: Let $E_{K}\left(\cdot \right)$ and $D_{K}\left(\cdot \right)$ denote symmetric encryption and decryption under key *K*. We assume that the symmetric encryption scheme is an authenticated encryption with associated data (AEAD) scheme providing confidentiality and ciphertext integrity.

Elliptic curve cryptography (ECC): Let G be an EC group of prime order *q* with generator *G*. Each device $D_{i}$ possesses a classical key pair $\left(d_{i},Q_{i}\right)$, where $d_{i}\in Z_{q}$ is the private key and $Q_{i}=d_{i}G$ is the corresponding public key. We assume that the Computational Diffie–Hellman (CDH) problem in G is hard and that the signature scheme derived from ECC is existentially unforgeable under chosen message attacks (EUF-CMA).

PQ key encapsulation mechanism (KEM): Each device also possesses a PQ key pair $\left(sk_{i},pk_{i}\right)$. The encapsulation algorithm takes as input a public key $pk_{j}$ and outputs a pair (e,K), where *e* is the ciphertext and *K* is a shared secret. The decapsulation algorithm takes $\left(e,sk_{j}\right)$ and outputs the same shared secret *K*. We assume that the KEM is secure under indistinguishability against chosen ciphertext attacks (IND-CCA).

Hash function: A cryptographic hash function H(·) is modeled as a random oracle in the security analysis.

### 3.3. Protocol Participants and Sessions

Each device can participate in multiple concurrent protocol executions, referred to as sessions. A session is an instance of the protocol executed by a device with the intention of establishing a shared session key with another device.

We denote by $\Pi_{i}^{s}$ the *s*th session executed by device $D_{i}$. Each session maintains the following state information:

- The identity of the peer device;
- The exchanged protocol messages;
- Locally generated randomness;
- Intermediate cryptographic values;
- The resulting session key SK (if successfully established).

Two sessions are said to be partner sessions if they have matching transcripts and derive the same session key.

### 3.4. Adversary Model (Informal)

We consider a probabilistic polynomial-time adversary A that has full control over the communication channel. The adversary can eavesdrop, intercept, modify, and inject messages between devices. Furthermore, A may attempt to impersonate legitimate devices or replay previously observed messages.

In addition, the adversary may obtain long-term secret keys of devices through corruption and may attempt to exploit stored communication transcripts in a future quantum setting. However, we assume that the distributed storage layer correctly returns the stored public key material and that onboarding ensures the authenticity of registered keys.

The formal security model and adversary capabilities will be defined in Section 5 using a Real-Or-Random (ROR) framework.

### 3.5. Security Goals

The proposed protocol aims to achieve the following security properties:

- Mutual authentication: Both participating devices are assured of each other’s identity.
- Session key confidentiality: The established session key remains indistinguishable from random to any adversary.
- Integrity and replay protection: Protocol messages cannot be modified or reused without detection.
- Conditional perfect forward secrecy: Compromise of long-term keys does not reveal past session keys, under the assumed threat model.
- PQ confidentiality: Session key secrecy remains secure against future quantum adversaries due to the use of PQ key encapsulation.

## 4. Proposed Protocol

The proposed scheme consists of two phases: (i) an onboarding phase, in which devices register their public key material in a distributed storage system, and (ii) a runtime AKA phase, in which two devices establish a shared session key.

All random values are assumed to be sampled uniformly from their respective domains. The hash function H(·) is modeled as a random oracle. The signature mechanism used in the protocol follows a Schnorr-type construction over the EC group.

### 4.1. Onboarding Phase

During the onboarding phase, each device $D_{i}$ registers its identity and public key material in the distributed storage system.

More specifically, device $D_{i}$ provides:

- Its identity $ID_{i}$;
- Its ECC public key $Q_{i}=d_{i}G$;
- Its PQ public key $pk_{i}$;
- Optional metadata (e.g., capabilities, manufacturer information).

During the onboarding phase, a trusted authority verifies the legitimacy of device $D_{i}$ based on predefined policies (e.g., manufacturer credentials or security requirements). Upon successful verification, the device’s public key material and metadata are packaged into a data object and stored in a distributed storage system, such as IPFS.

A content identifier $CID_{i}$ referencing the stored key material is generated and made publicly accessible via the distributed storage network. The content identifier $CID_{i}$ is derived as a cryptographic hash of this data object, ensuring integrity and immutability. The trusted authority either directly publishes or authorizes the publication of this content, thereby establishing a binding between the device identity and its public key material. Optionally, the trusted authority can digitally sign the stored data object, allowing devices to explicitly verify that the content was approved during onboarding.

Devices retrieving $\left(CID_{i},Q_{i},pk_{i}\right)$ can verify the integrity of the data through the content-addressing mechanism and rely on the onboarding process for authenticity.

We assume that the onboarding phase ensures the authenticity and integrity of the stored public key material. During the runtime protocol execution, devices can retrieve $\left(CID_{i},Q_{i},pk_{i}\right)$ through the distributed storage and verify its consistency. After onboarding, the trusted authority is no longer involved in the authentication process, ensuring that the protocol operates without an online TTP.

### 4.2. AKA Phase

The AKA phase is executed between two devices $D_{1}$ and $D_{2}$ that wish to establish a shared session key.

Initialization.

Device $D_{2}$ broadcasts its identifier and public key information:

$$M_{0}=\left\{CID_{2},Q_{2},pk_{2}\right\}.$$

Upon receiving $M_{0}$, device $D_{1}$ treats the included values $\left(Q_{2},pk_{2}\right)$ as untrusted until the corresponding record linked to $CID_{2}$ has been retrieved from the distributed storage and successfully verified. Only after this consistency check succeeds are the retrieved public keys accepted as authentic. We assume that this lookup returns authentic and untampered data.

Step 1 (from $D_{1}$ to $D_{2}$).  

After verifying the public key material of $D_{2}$, device $D_{1}$ initiates the AKA procedure by generating a PQ shared secret. To this end, it applies the encapsulation algorithm on the public key $pk_{2}$ of $D_{2}$, resulting in a ciphertext and a shared secret $\left(e_{a},K_{a}\right)\leftarrow \mathrm{Encaps}\left(pk_{2}\right)$.

Next, $D_{1}$ samples a random value $r_{a}\overset{\$}{\leftarrow }Z_{q}$ and computes the corresponding EC point $R_{a}=r_{a}G$. Using this value, $D_{1}$ constructs a Schnorr-type signature with a subtraction convention to authenticate its contribution. Note that the adopted subtraction-based Schnorr formulation is algebraically equivalent to the standard Schnorr signature construction and therefore inherits the same EUF-CMA security guarantees. Specifically, it computes a hash value $h_{a}=H(R_{a}\|e_{a}\left\|TS_{a}\right)$, where $TS_{a}$ denotes a timestamp ensuring freshness, and then computes the signature component $s_{a}=r_{a}-h_{a}d_{1}$. The pair $\left(R_{a},s_{a}\right)$ serves as a signature on the encapsulation output and associated context.

Subsequently, $D_{1}$ encrypts its identity, public key material, and signature parameters using the derived symmetric key $K_{a}$, resulting in the ciphertext

$$C_{a}=E_{K_{a}}\left(CID_{1},Q_{1},pk_{1},s_{a},R_{a},TS_{a}\right).$$

Finally, $D_{1}$ transmits the message $M_{1}=\left\{C_{a},e_{a},TS_{a}\right\}$ to $D_{2}$.

Step 2 (processing at $D_{2}$).  

Upon receiving the message $M_{1}$, device $D_{2}$ first reconstructs the shared secret $K_{a}$ by applying the decapsulation algorithm to the received encapsulation $e_{a}$, i.e., $K_{a}\leftarrow \mathrm{Decaps}\left(e_{a},sk_{2}\right)$. Using this key, it decrypts the ciphertext $C_{a}$ to recover the tuple $\left(CID_{1},Q_{1},pk_{1},s_{a},R_{a},TS_{a}\right)$.

Device $D_{2}$ then verifies the freshness of the message by checking the timestamp $TS_{a}$. It proceeds by retrieving the public key material corresponding to $CID_{1}$ from the distributed storage and verifying its consistency. The authenticity of $D_{1}$ is then established by verifying the Schnorr signature: $D_{2}$ recomputes $h_{a}=H(R_{a}\|e_{a}\left\|TS_{a}\right)$ and checks whether the equation $s_{a}G=R_{a}-h_{a}Q_{1}$ holds. If any of these checks fail, the protocol execution is aborted.

If all verifications succeed, $D_{2}$ continues by generating its own contribution to the session key. It computes $\left(e_{b},K_{b}\right)\leftarrow \mathrm{Encaps}\left(pk_{1}\right)$ using the public key of $D_{1}$. It then samples a fresh random value $r_{b}\overset{\$}{\leftarrow }Z_{q}$ and computes $R_{b}=r_{b}G$.

Using both shared secrets and the Diffie–Hellman component, $D_{2}$ derives the session key as

$$SK=H(K_{a}\|K_{b}\|r_{b}R_{a}).$$

To authenticate its contribution, $D_{2}$ computes $h_{b}=H(R_{b}\|e_{b}\|TS_{b}\left\|SK\right)$ and constructs the signature component $s_{b}=r_{b}-h_{b}d_{2}$.

Finally, $D_{2}$ encrypts the signature and timestamp under $K_{b}$, resulting in

$$C_{b}=E_{K_{b}}\left(s_{b},R_{b},TS_{b}\right),$$

and sends the message $M_{2}=\left\{C_{b},e_{b},TS_{b}\right\}$ to $D_{1}$.

Step 3 (processing at $D_{1}$).  

Upon receiving $M_{2}$, device $D_{1}$ recovers the shared secret $K_{b}$ by computing $K_{b}\leftarrow \mathrm{Decaps}\left(e_{b},sk_{1}\right)$ and decrypts the ciphertext $C_{b}$ to obtain $\left(s_{b},R_{b},TS_{b}\right)$. It verifies the freshness of the message by checking $TS_{b}$.

Next, $D_{1}$ derives the session key as

$$SK=H(K_{a}\|K_{b}\|r_{a}R_{b}).$$

It then recomputes the hash value $h_{b}=H(R_{b}\|e_{b}\|TS_{b}\left\|SK\right)$ and verifies the signature by checking whether $s_{b}G=R_{b}-h_{b}Q_{2}$. If the verification succeeds, $D_{1}$ accepts the session and outputs the session key SK.

Correctness.

The correctness of the protocol follows from the equality $r_{a}R_{b}=r_{b}R_{a}$, which ensures that both parties derive the same Diffie–Hellman component. Consequently, both devices compute the same session key:

$$SK=H(K_{a}\|K_{b}\|r_{a}R_{b}).$$

**Figure 1:**
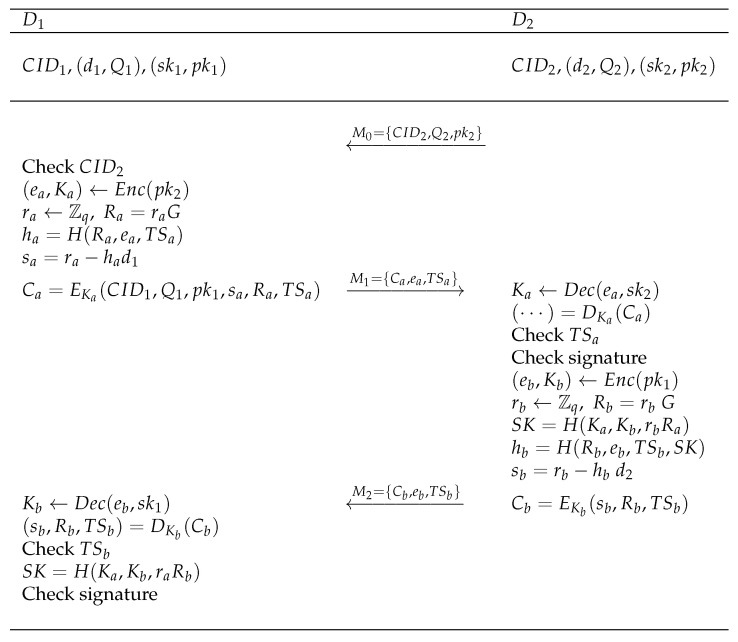
Proposed decentralized D2D AKA protocol.

Figure 1 illustrates the message flow and main cryptographic operations of the proposed protocol.

## 5. Security Analysis

In this section, we analyze the security of the proposed protocol from both an informal and a formal perspective. We first discuss how the protocol satisfies the main security requirements under the adopted system assumptions. Next, we present a Real-Or-Random (ROR) security model for session key indistinguishability and provide a proof sketch showing that the protocol is secure under the hardness of the underlying cryptographic assumptions.

### 5.1. Informal Security Analysis

#### 5.1.1. Mutual Authentication

Mutual authentication is achieved through the joint use of authenticated public key discovery and Schnorr-type signatures. Before accepting a peer, each device retrieves the corresponding public key material from the distributed storage and checks its consistency with the received identifier. Assuming that the onboarding process correctly binds device identities to their public keys and that the distributed storage returns authentic records, a malicious party cannot successfully replace public key material without being detected.

In addition, each party proves possession of its ECC private key by generating a valid Schnorr-type signature. In the first protocol message, $D_{1}$ signs the encapsulation output $e_{a}$ and the timestamp $TS_{a}$, while in the second protocol message, $D_{2}$ signs $e_{b}$, $TS_{b}$, and the derived session key SK. Therefore, an adversary that does not know the corresponding long-term private key cannot impersonate either participant except with negligible probability under the EUF-CMA security of the signature scheme.

#### 5.1.2. Session Key Confidentiality

The confidentiality of the established session key follows from the fact that it is derived as

$$SK=H(K_{a}\|K_{b}\|r_{a}R_{b}),$$

where $K_{a}$ and $K_{b}$ are obtained through PQ key encapsulation and $r_{a}R_{b}=r_{b}R_{a}$ is an EC Diffie–Hellman component.

The values $K_{a}$ and $K_{b}$ can only be recovered by the intended decapsulating parties holding $sk_{2}$ and $sk_{1}$, respectively. Hence, an adversary observing the protocol messages cannot reconstruct these values unless it breaks the IND-CCA security of the underlying PQ KEM. Moreover, even if one of the two encapsulated secrets were compromised, the session key would still depend on the remaining secret and the ephemeral Diffie–Hellman contribution. Since the hash function is modeled as a random oracle, the resulting session key is computationally indistinguishable from a random value to any efficient adversary.

#### 5.1.3. Integrity and Replay Protection

Message integrity is protected at two levels. First, the encrypted payloads $C_{a}$ and $C_{b}$ are protected by symmetric encryption under keys derived from the KEM outputs, providing both confidentiality and integrity protection, such that modified ciphertexts are rejected during authenticated decryption. Second, the Schnorr-type signatures bind the authenticated message components to the sender’s long-term ECC key.

Replay protection is achieved through timestamps $TS_{a}$ and $TS_{b}$. A replayed message is rejected because its timestamp falls outside the accepted freshness window. In addition, the message flow is bound to fresh KEM encapsulations and fresh ephemeral randomness, so replaying previously observed messages does not lead to the acceptance of a fresh session.

#### 5.1.4. Resistance to Impersonation and Man-in-the-Middle Attacks

An impersonation attack requires the adversary to either forge a valid signature under $Q_{1}$ or $Q_{2}$, or to substitute public key material during lookup. Under the onboarding and authenticated lookup assumptions, and assuming EUF-CMA security of the ECC signature mechanism, such impersonation succeeds only with negligible probability.

A man-in-the-middle adversary controlling the communication channel can intercept, delay, or modify messages, but cannot complete the protocol successfully unless it can both recover the KEM-derived keys and generate valid signatures. Since the second message authenticates the derived session key itself through $h_{b}=H(R_{b}\|e_{b}\|TS_{b}\left\|SK\right)$, the adversary cannot desynchronize the two parties into accepting different session keys.

#### 5.1.5. Availability and Denial-of-Service Considerations

As in most authentication protocols, cryptographic mechanisms alone cannot completely prevent denial-of-service attacks. In particular, an adversary may still flood a target device with malformed or bogus requests. However, the proposed protocol limits the attack surface in two ways. First, public key validation and freshness checking are performed before accepting a session. Second, the protocol consists of only two message rounds and does not require long state retention before validation. As a result, invalid sessions can be discarded early, reducing unnecessary computational and storage overhead. In practice, additional protections, such as rate limiting, anomaly detection, or admission control, should be deployed alongside the protocol.

#### 5.1.6. Conditional Perfect Forward Secrecy

The protocol provides conditional perfect forward secrecy under the current threat model. Specifically, if the long-term keys $\left(d_{1},sk_{1}\right)$ and $\left(d_{2},sk_{2}\right)$ are compromised after completion of a session, previously established session keys remain protected as long as the ephemeral values $r_{a}$ and $r_{b}$ have been erased and the adversary cannot solve the underlying Diffie-Hellman (DH) problem in the EC group during the session lifetime. If the long-term PQ KEM secret keys are later exposed, previously recorded encapsulations may be decapsulated, and the remaining protection of past session keys therefore depends on the secrecy of the erased ephemeral ECC values and the hardness of the classical DH problem.

This guarantee is conditional because the authentication layer relies on ECC. In a future large-scale quantum setting, the EC Diffie–Hellman component would no longer provide forward secrecy against a quantum-capable adversary. Nevertheless, the protocol still protects long-term confidentiality against harvest-now-decrypt-later attacks through the inclusion of the PQ KEM-derived secrets in the session key derivation.

#### 5.1.7. PQ Confidentiality

The protocol is designed to protect session key confidentiality against future quantum adversaries. Even if an adversary stores all exchanged protocol messages today and obtains a cryptographically relevant quantum computer later, it still cannot recover the KEM-derived values $K_{a}$ and $K_{b}$ without breaking the PQ encapsulation mechanism. Since these values are included in the final session key derivation, past session confidentiality remains protected under the IND-CCA security of the PQ KEM.

It should be emphasized that this PQ guarantee applies primarily to confidentiality. The authentication mechanism remains ECC-based and therefore follows a classical security assumption. This design choice reflects the different temporal requirements of confidentiality and authentication, and is intended as a practical trade-off for constrained environments.

### 5.2. Formal Security Model

We now formalize session key security using a standard Real-Or-Random (ROR) model. Let $\Pi_{i}^{s}$ denote the *s*th session instance executed by device $D_{i}$. An adversary A is modeled as a probabilistic polynomial-time algorithm interacting with honest parties through the following queries.

- Execute$\left(\Pi_{i}^{s},\Pi_{j}^{t}\right)$: This query models passive attacks. The oracle returns the transcript of an honest execution between two partner sessions $\Pi_{i}^{s}$ and $\Pi_{j}^{t}$.
- Send$\left(\Pi_{i}^{s},m\right)$: This query models active attacks. The adversary sends a message *m* to session $\Pi_{i}^{s}$ and receives the protocol response generated according to the protocol specification.
- Reveal$\left(\Pi_{i}^{s}\right)$: If session $\Pi_{i}^{s}$ has accepted, this query returns the session key held by $\Pi_{i}^{s}$.
- CorruptECC$\left(D_{i}\right)$: returns the ECC authentication secret $d_{i}$, while CorruptPQ($D_{i}$) returns the PQ KEM secret key $sk_{i}$. This distinction allows separate modeling of authentication compromise and PQ confidentiality compromise.
- Test$\left(\Pi_{i}^{s}\right)$: This query can be asked once to a fresh session $\Pi_{i}^{s}$. The oracle flips a random bit b∈{0,1}. If b=1, it returns the real session key held by $\Pi_{i}^{s}$; otherwise, it returns a random string of the same length. At the end of the experiment, the adversary outputs a guess $b^{\prime }$.

Freshness

A session $\Pi_{i}^{s}$ is said to be fresh if all of the following conditions hold:

- $\Pi_{i}^{s}$ has accepted;
- Neither $\Pi_{i}^{s}$ nor its partner session has been queried via reveal;
- The long-term secrets of both parties were not corrupted before the session completed;
- The session under test is not trivially exposed through another partnered instance.

Additionally, a session is not considered fresh if the corresponding PQ KEM secret key was compromised through a CorruptPQ query before the test query.

ROR Advantage

The advantage of adversary A in the ROR experiment is defined as

$$Adv_{A}^{ROR}=\left|2\mathrm{Pr}[b^{\prime }=b]-1\right|.$$

The protocol is said to provide session key indistinguishability if $Adv_{A}^{ROR}$ is negligible for any PPT adversary A.

**Theorem** **1.** *Assume that: (i) the employed PQ KEM is IND-CCA secure, (ii) the Schnorr-type ECC signature scheme is EUF-CMA secure, (iii) the Computational Diffie–Hellman (CDH) problem in the EC group is hard, and (iv) the hash function H behaves as a random oracle. Then the proposed protocol achieves session key indistinguishability in the ROR model for all fresh sessions.*

**Proof.**  We prove the theorem through a sequence of games.

*Game $G_{0}$.* This is the real ROR experiment. Let the adversary’s success probability in this game be $\mathrm{Pr}[S_{0}]$.

*Game $G_{1}$.* In this game, we abort if the adversary successfully forges a valid Schnorr-type signature in either protocol direction without querying the corresponding honest signer. Any difference between $G_{0}$ and $G_{1}$ therefore implies an existential forgery against the ECC signature scheme. Hence,

$$|\mathrm{Pr}\left[S_{1}\right]-\mathrm{Pr}\left[S_{0}\right]|\leq Adv_{B_{1}}^{EUF-CMA},$$

for some efficient adversary $B_{1}$ against the signature scheme.

*Game $G_{2}$.* Next, we replace the KEM-derived secret $K_{a}$ in the test session with a uniformly random string independent of the encapsulation $e_{a}$. If the adversary can distinguish this modification, then one can construct an adversary $B_{2}$ that breaks the IND-CCA security of the PQ KEM. Therefore,

$$|\mathrm{Pr}\left[S_{2}\right]-\mathrm{Pr}\left[S_{1}\right]|\leq Adv_{B_{2}}^{IND-CCA}.$$

*Game $G_{3}$.* Similarly, we replace the second KEM-derived secret $K_{b}$ in the test session with an independent uniformly random string. Again, any non-negligible difference would contradict the IND-CCA security of the KEM. Thus,

$$|\mathrm{Pr}\left[S_{3}\right]-\mathrm{Pr}\left[S_{2}\right]|\leq Adv_{B_{3}}^{IND-CCA}.$$

Before proceeding to Game $G_{4}$, we clarify that the transition to the CDH-based argument is considered only after the KEM-derived values $K_{a}$ and $K_{b}$ in the tested fresh session have been replaced by independent random strings in Games $G_{2}$ and $G_{3}$. Therefore, the CDH reduction is not intended to model attacks in which the adversary obtains the PQ decapsulation secret keys and directly recovers the KEM outputs from recorded encapsulations. Such cases are excluded by the freshness definition for PQ confidentiality. Rather, Game $G_{4}$ captures the remaining distinguishing advantage associated with the ephemeral DH contribution once the KEM-derived components are already hidden.

*Game $G_{4}$.* In the final step, we replace the Diffie–Hellman term $r_{a}R_{b}=r_{b}R_{a}$ used in the test session with a random group element. If the adversary notices this change, then one can build an adversary $B_{4}$ that solves the CDH problem in the EC group. Hence,

$$|\mathrm{Pr}\left[S_{4}\right]-\mathrm{Pr}\left[S_{3}\right]|\leq Adv_{B_{4}}^{CDH}.$$

At this point, the session key in the test session is computed as

$$SK=H(K_{a}^{\prime }\|K_{b}^{\prime }\|Z^{\prime }),$$

where $K_{a}^{\prime }$ and $K_{b}^{\prime }$ are independent random strings and $Z^{\prime }$ is an independent random group element. Since *H* is modeled as a random oracle, the resulting session key is uniformly random from the adversary’s point of view unless it queries the exact input to the oracle. The probability of this event is negligible. Therefore,

$$\mathrm{Pr}\left[S_{4}\right]=\frac{1}{2}+negl\left(\lambda \right).$$

Combining the game transitions yields

$$Adv_{A}^{ROR}\leq 2Adv_{B_{1}}^{EUF-CMA}+2Adv_{B_{2}}^{IND-CCA}+2Adv_{B_{3}}^{IND-CCA}+2Adv_{B_{4}}^{CDH}+negl\left(\lambda \right),$$

which is negligible under the stated assumptions.    □

The above theorem shows that the proposed protocol provides session key indistinguishability for fresh sessions against a classical active adversary in the ROR model. The confidentiality contribution of the protocol is strengthened by the use of a PQ KEM, which protects against future quantum decryption of recorded protocol transcripts. At the same time, authentication and the ephemeral Diffie–Hellman contribution rely on classical EC assumptions. Consequently, the formal result should be interpreted as a hybrid guarantee: PQ protection for long-term confidentiality and classical hardness for authentication and conditional forward secrecy.

This interpretation is fully consistent with the intended threat model of the protocol, in which confidentiality must remain secure against future quantum adversaries, while authentication is required at protocol runtime under current computational assumptions.

## 6. Prototype Validation and Performance Analysis

### 6.1. Implementation Setup

To validate the correctness and executability of the proposed protocol, we developed a proof-of-concept software prototype that captures both phases of the scheme: the trusted onboarding phase and the runtime D2D AKA phase. The prototype was executed on a standard computing environment and is intended for functional validation rather than hardware-specific benchmarking. The prototype includes two main roles: (i) a trusted onboarding entity that verifies devices and publishes their public information, and (ii) IoT devices that execute the proposed authentication and key agreement procedure.

The prototype was implemented in Python 3.12. ECC-based operations were realized using the ecdsa library, post-quantum KEM functionality was implemented through liboqs, and symmetric encryption was realized using pycryptodomex. For decentralized public key discovery, the system interfaces with IPFS, which is used to store and retrieve device identifiers, public keys, and associated metadata generated during the onboarding phase.

At the implementation level, each device maintains its identity, ECC key pair, and PQ KEM key pair, and executes the protocol steps described in Section 4. The onboarding entity is responsible for validating whether a device satisfies the enrollment requirements and for binding the device identity to the published public key material. During protocol execution, a device first retrieves the peer public information through IPFS and then performs the two-message hybrid AKA exchange.

The goal of this prototype is not to provide cycle-accurate benchmarking on a specific hardware platform, but to demonstrate the feasibility of the protocol logic, message flow, and integration of the underlying cryptographic and decentralized storage components. Therefore, the performance evaluation in the following subsections follows a literature-based benchmarking methodology, using reported operation costs for the underlying ECC and post-quantum primitives on a representative lightweight platform. This allows a fair comparison with related schemes while keeping the implementation discussion aligned with the protocol contribution of this paper.

### 6.2. Computational Cost

Following the methodology used in [30], we focus on the dominant asymmetric cryptographic operations and neglect the comparatively small contribution of symmetric encryption, hashing, and point additions. In particular, we use the Raspberry Pi 4 benchmark setting adopted in [30], where the median costs are 0.23 ms for one EC point multiplication and, for Kyber, 0.08 ms for key generation, 0.12 ms for encapsulation, and 0.14 ms for decapsulation (see Table 2). This provides a uniform and fair basis for comparing representative PQ and hybrid schemes. Schemes [14,30] are selected as the main reference points, since both are KEM-based alternatives already discussed in the related-work section.

Based on the protocol descriptions, the dominant asymmetric costs can be summarized as follows. The scheme of [30] requires one encapsulation, two decapsulations, and four EC point multiplications. The scheme of [14] requires one Kyber key generation, two encapsulations, and two decapsulations. The proposed D2D protocol requires two encapsulations, two decapsulations, and six EC point multiplications. The resulting computational costs are shown in Table 3.

As expected, the proposed scheme is computationally heavier than [14], since the latter operates in a more structured setting and does not include the same level of decentralized mutual authentication. Compared with [30], the proposed protocol incurs a moderate increase in cost, mainly due to the additional EC operations required to authenticate both peers in a D2D environment without relying on pre-shared device-specific context. Nevertheless, the total cost remains below 2 ms on the Raspberry Pi 4 reference platform, which indicates that the protocol is still practical for lightweight IoT-class devices.

### 6.3. Communication Overhead

The communication overhead is dominated by the PQ material. Again following the parameterization used in [30], Kyber requires an 800-byte public key and a 768-byte ciphertext on the considered security level. In the proposed D2D protocol, the two KEM ciphertexts $e_{a}$ and $e_{b}$ already contribute 2×768=1536 bytes. In addition, two Kyber public keys appear on the D2D exchange path, contributing 2×800=1600 bytes. As a result, the PQ material alone contributes 3136 bytes, before accounting for ECC public values, signature components, timestamps, identifiers, and encryption overhead.

This communication cost is higher than in the partial-hybrid variant reported in [30], where a total of 896 bytes was obtained for the best Kyber-based mode, and also higher than the 1696-byte full-hybrid mode in the same paper. The reason is structural: in the present work, the protocol operates in a decentralized D2D setting without pre-installed peer-specific context, so both parties must explicitly exchange the key-establishment material needed for mutual authentication and confidentiality. This is the price paid for runtime autonomy and removal of the online trusted third party.

At the same time, the proposed architecture avoids certificate chains and repeated PKI artifacts during runtime. Hence, the role of the IPFS-assisted key discovery layer is not to reduce the raw byte size of PQ primitives, but to eliminate additional certificate-management overhead while preserving decentralized availability of authenticated public key material. The communication overhead is primarily determined by the intrinsic size of PQ primitives rather than inefficiencies in the protocol design.

### 6.4. Discussion

The performance analysis highlights the central trade-off of the proposed design. Compared with structured client–server or pre-contextualized protocols, the proposed D2D scheme incurs additional computation and communication costs because it supports decentralized peer authentication without an online trusted third party. However, this overhead remains reasonable in view of the stronger trust model and the inclusion of PQ confidentiality protection. In particular, the computational overhead is still modest on Raspberry Pi 4, whereas the communication overhead is mainly driven by the inherent size of current lattice-based KEM primitives rather than by inefficient protocol design.

## 7. Conclusions and Future Work

This paper presented a decentralized post-quantum authentication and key agreement protocol for device-to-device communication. The proposed scheme combines post-quantum key encapsulation with elliptic-curve-based authentication and leverages IPFS for distributed key management, enabling secure D2D communication without relying on an online trusted third party or pre-shared context.

A key design choice is the explicit separation between confidentiality and authentication requirements. Post-quantum primitives are used to protect against harvest-now-decrypt-later attacks, ensuring long-term confidentiality of exchanged data, while classical ECC-based signatures are retained for authentication due to their efficiency and the real-time nature of identity verification. This hybrid approach avoids the high overhead of post-quantum signatures while maintaining strong security guarantees under the current threat model.

The protocol achieves mutual authentication, resistance against active attacks, and session key confidentiality, as confirmed by the ROR-based security analysis. Although post-quantum primitives increase communication overhead, the computational cost remains practical for IoT-class devices, and the decentralized design eliminates certificate management and centralized infrastructure.

Overall, the proposed approach provides a practical and well-balanced solution for secure and quantum-resilient D2D communication in future IoT and 6G environments.

As future work, several directions can be explored. First, the integration of fully PQ authentication mechanisms could remove the remaining reliance on classical ECC. Second, optimizing communication overhead through more compact PQ primitives or compression techniques remains an important challenge. Third, a full implementation and evaluation on embedded hardware platforms would provide deeper insights into real-world deployment constraints. Finally, extending the protocol toward group-based D2D communication and integration with emerging 6G architectures represents a promising research direction.

## Figures and Tables

**Table 1:** Comparison with related D2D and IoT AKA schemes. (* PQ security refers to confidentiality protection through PQ key encapsulation, while authentication relies on classical cryptographic mechanisms).

Scheme	D2D	No Online TTP	No Pre-Shared Context	PQ-Secure	PFS	Decentralized Key Mgmt
[7]-2nd	✓	×	✓	×	×	×
[8]	✓	×	×	×	×	×
[14,15,16]	×	×	×	✓	×	×
[17]	✓	×	×	✓	×	×
[29]	×	×	×	✓	×	✓
[30]	×	✓	×	✓	✓	×
[36]	×	×	×	×	×	✓
This work	✓	✓	✓	✓ *	✓	✓

**Table 2:** Cost of dominant cryptographic operations on Raspberry Pi 4.

Operation	Time (ms)
ECC point multiplication (*M*)	0.23
Kyber key generation (*G*)	0.08
Kyber encapsulation (*E*)	0.12
Kyber decapsulation (*D*)	0.14

**Table 3:** Comparison of dominant computational costs on Raspberry Pi 4.

Scheme	Ops S-Side	Time S-Side (ms)	Ops R-Side	Time R-Side (ms)	Total (ms)
[30]	E+2M	0.58	2D+2M	0.74	1.32
[14]	G+E	0.20	E+2D	0.40	0.60
This	E+2M	0.58	E+2D+4M	1.32	1.90

## Data Availability

No external datasets were used in this study. The work is based on the design and evaluation of a Lightweight Hybrid Authentication and Key Agreement Protocol, and no publicly archived dataset was generated or analyzed.

## References

[1] Yang H., Li Z., Luo C., Wei B., Xu W. (2024). InaudibleKey2. 0: Deep Learning-Empowered Mobile Device Pairing Protocol Based on Inaudible Acoustic Signals. IEEE/ACM Trans. Netw..

[2] Khalfaoui S., Leneutre J., Villard A., Ma J., Urien P. (2021). Security Analysis of Out-of-Band Device Pairing Protocols: A Survey. Wirel. Commun. Mob. Comput..

[3] Bernstein D.J., Lange T. (2017). Post-quantum cryptography. Nature.

[4] Baek J., Newmarch J., Safavi-Naini R., Susilo W. A survey of identity-based cryptography. Proceedings of the Australian Unix Users Group Annual Conference.

[5] Ali M.S., Dolui K., Antonelli F. IoT data privacy via blockchains and IPFS. Proceedings of the Seventh International Conference on the Internet of Things.

[6] Conti M., Kumar G., Nerurkar P., Saha R., Vigneri L. (2022). A survey on security challenges and solutions in the IOTA. J. Netw. Comput. Appl..

[7] Braeken A., Liyanage M., Jurcut A.D. (2019). Anonymous lightweight proxy based key agreement for IoT (ALPKA). Wirel. Pers. Commun..

[8] Braeken A. (2018). PUF based authentication protocol for IoT. Symmetry.

[9] Chatterjee U., Chakraborty R.S., Mukhopadhyay D. (2017). A PUF-based secure communication protocol for IoT. ACM Trans. Embed. Comput. Syst. (TECS).

[10] (2020). Security Architecture and Procedures fo 5G System (Release 16).

[11] Braeken A., Liyanage M., Kumar P., Murphy J. (2019). Novel 5G authentication protocol to improve the resistance against active attacks and malicious serving networks. IEEE Access.

[12] Braeken A. (2020). Symmetric key based 5G AKA authentication protocol satisfying anonymity and unlinkability. Comput. Netw..

[13] Munilla J., Burmester M., Barco R. (2021). An enhanced symmetric-key based 5G-AKA protocol. Comput. Netw..

[14] Damir M.T., Meskanen T., Ramezanian S., Niemi V. (2022). A beyond-5G authentication and key agreement protocol. Proceedings of the International Conference on Network and System Security.

[15] Rossi Figlarz G., Passuelo Hessel F. (2024). Enhancing the 5G-AKA Protocol with Post-quantum Digital Signature Method. Proceedings of the International Conference on Advanced Information Networking and Applications.

[16] Joudah R.H., Manaa M.E. (2024). A New Approach to Improving the Security of the 5G-AKA Using Crystals-Kyber Post-Quantum Technologies and ASCON Algorithm. Int. Inf. Eng. Technol. Assoc..

[17] Selvakumar S., Ahilan A., Ben Sujitha B., Muthukumaran N. (2024). Crystals kyber cryptographic algorithm for efficient IoT D2d communication. Wirel. Netw..

[18] Babu P.R., Kumar S.A., Reddy A.G., Das A.K. (2024). Quantum secure authentication and key agreement protocols for IoT-enabled applications: A comprehensive survey and open challenges. Comput. Sci. Rev..

[19] Chikouche N., Cayrel P.L., Mboup E.H.M., Boidje B.O. (2019). A privacy-preserving code-based authentication protocol for Internet of Things. J. Supercomput..

[20] Zhang S., Du X., Liu X. (2023). A novel and quantum-resistant handover authentication protocol in IoT environment. Wirel. Netw..

[21] Rana S., Mishra D. (2021). Lattice-based key agreement protocol under ring-LWE problem for IoT-enabled smart devices. Sādhanā.

[22] Li Z., Wang D. (2019). Achieving one-round password-based authenticated key exchange over lattices. IEEE Trans. Serv. Comput..

[23] Al-Saggaf A.A., Sheltami T., Alkhzaimi H., Ahmed G. (2023). Lightweight two-factor-based user authentication protocol for iot-enabled healthcare ecosystem in quantum computing. Arab. J. Sci. Eng..

[24] Wei G., Fan K., Zhang K., Wang H., Li H., Yang Y. (2023). Quantum-Safe Lattice-Based Certificateless Anonymous Authenticated Key Agreement for Internet of Things. IEEE Internet Things J..

[25] Basu S., Seyhan K., Islam S.H., Akleylek S. (2023). MLWR-2PAKA: A hybrid module learning with rounding-based authenticated key agreement protocol for two-party communication. IEEE Syst. J..

[26] Braeken A., Yadav A.K. (2025). Cryptanalysis of Post-Quantum Security Schemes based on the Hardness of the Inhomogeneous Small Integer Solution (ISIS) problem. IEEE Trans. Consum. Electron..

[27] Islam S.H. (2020). Provably secure two-party authenticated key agreement protocol for post-quantum environments. J. Inf. Secur. Appl..

[28] Aujla G.S., Chaudhary R., Kaur K., Garg S., Kumar N., Ranjan R. (2018). SAFE: SDN-assisted framework for edge–cloud interplay in secure healthcare ecosystem. IEEE Trans. Ind. Inform..

[29] Abood E.W., Yassin A.A., Abduljabbar Z.A., Nyangaresi V.O., Ali A.H. (2025). Provably lightweight and secure IoHT scheme with post-quantum cryptography and fog computing: A comprehensive scheme for healthcare system. MethodsX.

[30] Braeken A. (2025). Flexible hybrid post-quantum bidirectional multi-factor authentication and key agreement framework using ECC and KEM. Future Gener. Comput. Syst..

[31] Agyekum K.O.B.O., Xia Q., Sifah E.B., Cobblah C.N.A., Xia H., Gao J. (2021). A proxy re-encryption approach to secure data sharing in the internet of things based on blockchain. IEEE Syst. J..

[32] Mishra R.A., Kalla A., Braeken A., Liyanage M. (2023). Blockchain regulated verifiable and automatic key refreshment mechanism for IoT. IEEE Access.

[33] Hewa T., Bracken A., Ylianttila M., Liyanage M. (2020). Blockchain-based automated certificate revocation for 5G IoT. Proceedings of the ICC 2020—2020 IEEE International Conference on Communications (ICC).

[34] Garba A., Khoury D., Balian P., Haddad S., Sayah J., Chen Z., Guan Z., Hamdan H., Charafeddine J., Al-Mutib K. (2023). LightCERT4IoTs: Blockchain-based lightweight certificates authentication for IoT applications. IEEE Access.

[35] Yang D., Yoo S., Doh I., Chae K. (2021). Selective blockchain system for secure and efficient D2D communication. J. Netw. Comput. Appl..

[36] Liu S., Chen L., Yu H., Gao S., Fang H. (2023). BP-AKAA: Blockchain-enforced Privacy-preserving Authentication and Key Agreement and Access Control for IIoT. J. Inf. Secur. Appl..

[37] Yao W., Gorlewski N., Deek F.P., Wang G. (2024). Considerations for Decision Makers and Developers Toward the Adoption of Decentralized Key Management Systems Technology in Emerging Applications. Computer.

[38] Benrebbouh C., Mansouri H., Cherbal S., Messai M.L., Pathan A.S.K. (2026). A survey of quantum and blockchain security solutions for IoT-based Energy Internet. Comput. Electr. Eng..

[39] Chen H., Wang W., Duan Y. (2025). A dual blockchain-based privacy-preserving authentication scheme for Vehicular Ad Hoc Networks. Comput. Netw..

[40] Tan H., Wang M., Shen J., Vijayakumar P., Moh S., Wu Q.J. (2025). Blockchain-assisted conditional anonymous authentication and adaptive tree-based group key agreement for VANETs. IEEE Trans. Dependable Secur. Comput..

[41] Ren Y., Li X., Sun S.F., Yuan X., Zhang X. (2021). Privacy-preserving batch verification signature scheme based on blockchain for vehicular ad-hoc networks. J. Inf. Secur. Appl..

